# Contribution of Cation Addition to MnO_*2*_ Nanosheets on Stable Co_*3*_O_*4*_ Nanowires for Aqueous Zinc-Ion Battery

**DOI:** 10.3389/fchem.2020.00793

**Published:** 2020-09-23

**Authors:** Nengze Wang, Gaochen Yang, Yi Gan, Houzhao Wan, Xu Chen, Cong Wang, Qiuyang Tan, Jie Ji, Xiaojuan Zhao, Pengcheng Liu, Jun Zhang, Xiaoniu Peng, Hanbin Wang, Yi Wang, Guokun Ma, Peter A. van Aken, Hao Wang

**Affiliations:** ^1^Hubei Key Laboratory of Ferro and Piezoelectric Materials and Devices, Faculty of Physics and Electronic Science, Hubei University, Wuhan, China; ^2^Max Planck Institute for Solid State Research, Stuttgart, Germany

**Keywords:** zinc-ion battery, MnO_2_, nanosheets, cation additives, flexible battery

## Abstract

Zinc-based electrochemistry attracts significant attention for practical energy storage owing to its uniqueness in terms of low cost and high safety. In this work, we propose a 2.0-V high-voltage Zn–MnO_2_ battery with core@shell Co_3_O_4_@MnO_2_ on carbon cloth as a cathode, an optimized aqueous ZnSO_4_ electrolyte with Mn^2+^ additive, and a Zn metal anode. Benefitting from the architecture engineering of growing Co_3_O_4_ nanorods on carbon cloth and subsequently deposited MnO_2_ on Co_3_O_4_ with a two-step hydrothermal method, the binder-free zinc-ion battery delivers a high power of 2384.7 W kg^−1^, a high capacity of 245.6 mAh g^−1^ at 0.5 A g^−1^, and a high energy density of 212.8 Wh kg^−1^. It is found that the Mn^2+^ cations are *in situ* converted to Mn_3_O_4_ during electrochemical operations followed by a phase transition into electroactive MnO_2_ in our battery system. The charge-storage mechanism of the MnO_2_-based cathode is Zn^2+^/Zn and H^+^ insertion/extraction. This work shines light on designing multivalent cation-based battery devices with high output voltage, safety, and remarkable electrochemical performances.

## Introduction

The ever-growing demands for electrical energy storage have stimulated the pursuit of advanced energy sources [batteries (Zhang H. et al., 2019), supercapacitors (Gan et al., 2020), photoelectrocatalysis (Wang et al., 2016; Shu et al., 2017)] with high energy density and high power density as well as high cycle lifetime (Pan et al., 2016; Ming et al., 2019; Zhang N. et al., 2019; Blanc et al., 2020). Li-ion batteries (LIBs) have become a protagonist due to their excellent comprehensive performances. However, lithium would present a long-term risk of shortage, since it is not quite abundant and even predicted to be regarded as the gold in the coming century (Li et al., 2019; Li C. et al., 2020). Zn-ion batteries (ZIBs), as a pronounced alternative, are receiving increased attention due to their merits of low cost, high safety, and high eco-efficiency. The ZIB device, in particular, consists of a zinc-metal anode, an aqueous electrolyte in majority and a cathode for accommodation of Zn ions. Among these components, the zinc anode has intrinsic merits of high theoretical capacity of 820 mAh g^−1^ and a low redox potential of −0.76 V vs. a standard hydrogen electrode (SHE). The aqueous electrolyte not only is safe and cheap but also has typically two times higher conductivity than their organic counterparts and hence may satisfy the high capacity and high power demands (Chae et al., 2017; Zhang et al., 2018; Yu et al., 2019). Multivalent aqueous ZIBs allow multiple electron transfer during electrochemical reactions instead of single electron transfer in LIBs (Fang et al., 2018; Verma et al., 2019).

Similar to LIBs, transition-metal oxides [Mn-based materials (Zhang H. et al., 2020; Zhang M. et al., 2020), V-based materials (Fang et al., 2019; Zhang L. et al., 2020), and Mo-based materials (He et al., 2019)], Prussian blue analogs (Trocoli and La Mantia, 2015), and organic materials (Zhang H. et al., 2020) are attractive cathode-material candidates in ZIBs, particularly manganese dioxide (MnO_2_), owing to its low cost and low toxicity. MnO_2_ exists in various crystallographic polymorphs, i.e., the α-, β-, γ-, δ-, λ-, and ε-phase, which are composed of MnO_6_ octahedral units, where each Mn^4+^ cation is coordinated by six oxygen atoms (Song et al., 2018; Han et al., 2020). These fundamental structural units are interconnected to each other by edges and/or corners, forming various crystalline structures that correspond to these different polymorphs. Among them, layered-type δ-MnO_2_ demonstrates decent electrochemical performances vs. zinc, due to its relatively large interlayer distance (~7.0 Å) (Hou et al., 2020). The Kim's group presented a δ-MnO_2_ ZIB with 252 mAh g^−1^ high capacity at 83 mA g^−1^, which maintained 112 mAh g^−1^ capacity after cycling for 100 cycles. Increasing the current density to 666 mA g^−1^ and 1,333 mA g^−1^, the capacities become 92 and 30 mAh g^−1^ (Alfaruqi et al., 2015). Zhi's group revealed a Na^+^ and H_2_O pre-intercalated δ-MnO_2_ ZIB with a high capacity of 278 mAh g^−1^ at 1 C, which maintained a capacity of 106 mAh g^−1^ when increasing the current to 1 C, and this ZIB keeps 98% capacity after cycling for 10,000 cycles (Wang et al., 2019). Guo et al. showed an ultrathin δ-MnO_2_ nanosheet ZIB, exhibiting a reversible capacity of 133 mAh g^−1^ at the current density of 100 mA g^−1^, which maintained 86 mAh g^−1^ when increasing the current density to 500 mA g^−1^ (Guo C. et al., 2019).

To construct a more advanced zinc-ion battery device, optimizing its structure of both the cathode electroactive materials and the electrolyte is necessary (Cai et al., 2018; Guo S. et al., 2019; Pan et al., 2019; Tan et al., 2020; Wang et al., 2020). Highly conductive and high-surface-area Co_3_O_4_ nanowires were firstly grown on carbon cloth, followed by growing a layered structure of δ-MnO_2_ nanosheets on Co_3_O_4_ by a two-step hydrothermal method. Mn^2+^ and Co^2+^ additives were added into an aqueous ZnSO_4_ electrolyte to optimize the cation storage within δ-MnO_2_. The aqueous zinc ion battery demonstrates outstanding electrochemical performances, and the charge storage mechanism and the effects of the electrolyte additive contribution were systemically investigated.

## Experimental

### Material

Cobalt chloride hexahydrate (CoCl_2_·H_2_O, >99.5%), urea [CO(NH_2_)_2_, >99.5%], potassium permanganate (KMnO_4_, >99.0%), carbon cloth (CC), zinc sulfate heptahydrate (ZnSO_4_·7H_2_O, 99.0%), cobalt sulfate heptahydrate (CoSO_4_·7H_2_O, >99.0%), and manganese sulfate monohydrate (MnSO_4_·H_2_O, >99.99%) were purchased from Sinopharm Chemical Reagent. All chemicals were used as received.

### Synthesis

#### Preparation of Co_3_O_4_ Nanowires on Carbon Cloth

Co_3_O_4_ nanowires were synthesized by a hydrothermal method on the surface of carbon cloth, which is used as substrate for growing all cathode materials throughout this manuscript. As for electrochemical measurements, carbon cloth serves as a flexible current collector. In a typical synthesis process, 9 mmol CoCl_2_·6H_2_O and 4 mmol urea were dissolved into 60 ml of deionized (DI) water by stirring for 2 h. Then, the solution was transferred into a 100-ml Teflon liner with a 3 × 4 cm carbon cloth substrate. After hydrothermal reaction at 120°C for 10 h, the Co_3_O_4_ nanowire precursors were formed on carbon cloth, which was cleaned with DI water, dried in an oven at 70°C, and then annealed in a tubular furnace at 350°C for 2 h with a heating rate of 2°C min^−1^.

#### Deposition of δ-MnO_2_ Nanosheets on the Surface of Co_3_O_4_ Nanowires

The as-synthesized Co_3_O_4_ nanowire/CC was transferred into a Teflon liner with 60 ml of 5 mM KMnO_4_ solution. After a second hydrothermal reaction at 160°C for 4 h, the δ-MnO_2_ nanosheets were deposited on the surface of Co_3_O_4_ nanowires, forming core-shell Co_3_O_4_@δ-MnO_2_ on CC substrate, which was then washed with DI water and dried at 70°C before electrochemical measurements.

### Characterizations

X-ray diffraction patterns were collected with a Bruker D8 diffractometer with Cu Kα radiation. SEM characterization was accomplished with a JEOL JSM-7100F, and TEM analysis was performed using a JEOL ARM 200F microscope equipped with a cold field emission electron source, an image Cs corrector (CEOS GmbH), and a Gatan image filter operated at 200 kV. X-ray photoelectron spectroscopy (XPS, Thermo Escalab) was used to identify elements and valence states, to determine the relative content of elements, to understand the interaction between central ions and coordination atoms and charge distribution, and to analyze the structure of compounds.

### Electrochemical Testing

#### Punch Type Cell Packaging and Testing

The as-synthesized Co_3_O_4_@δ-MnO_2_ on CC was sliced into a disk with a diameter of 1.2 cm and used as the cathode, 2 M ZnSO_4_ aqueous solution was used as the electrolyte, and a zinc foil (~0.07 mm thickness) was used as the anode, to encapsulate a punch type ZIB cell. In addition, MnSO_4_ or CoSO_4_ additive was added into the electrolyte with the cation (Mn^2+^ or Co^2+^) concentration of 0.2 M. The mass loading of the cathode was about 2.5 mg, including Co_3_O_4_, MnO_2_, and carbon cloth. Cyclic voltammetry (CV), galvanostatic charge–discharge (GCD), cycle life, and other electrochemical performance were tested by CHI760E electrochemical workstation and Neware battery test system. All electrochemical tests were performed at room temperature.

## Results and Discussions

**Figure 1:**
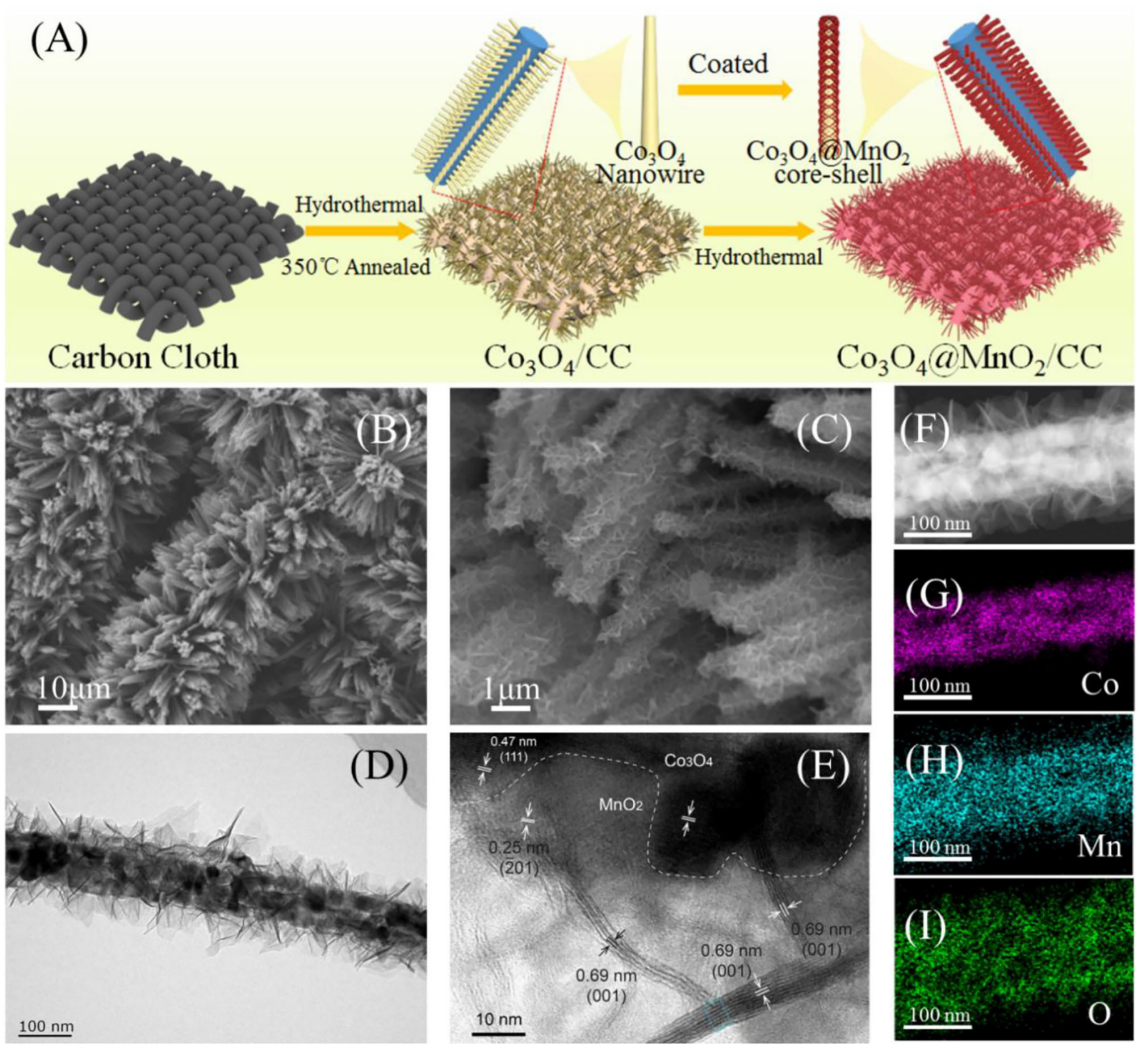
**(A)** Formation mechanism of Co_3_O_4_ nanowires@MnO_2_ nanosheets/CC. **(B,C)** Low-magnification and high-magnification SEM images of Co_3_O_4_@MnO_2_/CC. **(D,E)** Low-magnification and high-magnification TEM images of Co_3_O_4_@MnO_2_. **(F–I)** STEM-EDX elemental mapping of a single Co_3_O_4_@MnO_2_ nanowire.

The formation of core@shell Co_3_O_4_@MnO_2_ on carbon cloth is illustrated in Figure 1A. Alkaline cobalt carbonate nanowires were firstly grown on carbon cloth by a hydrothermal method, which were converted into Co_3_O_4_ nanowire arrays by a following thermal treatment. δ-MnO_2_ nanosheets were deposited on the surface of Co_3_O_4_ by a second hydrothermal reaction to form core@shell Co_3_O_4_@MnO_2_. SEM imaging was used to monitor its morphology evolution after hydrothermal reactions. Figures S1A,B show pristine Co_3_O_4_ grown on carbon cloth and demonstrate its needle shape. After coping with MnO_2_, core-shell nanowires were obtained, as shown in the low- and high-magnification SEM images of Figures 1B,C, respectively. TEM imaging was used to characterize the microstructures of as-synthesized Co_3_O_4_@MnO_2_, as shown in Figure 1D, which indicates that MnO_2_ are thin, with nanosheet shape and uniformly covering the surface of Co_3_O_4_. Figure 1E is a high-resolution TEM (HRTEM) image of a surface area of Co_3_O_4_@MnO_2_, in which the 0.47 nm lattice can be assigned to the (111) plane of Co_3_O_4_ and the 0.25 nm lattice is assigned to the (−201) plane of δ-MnO_2_. In addition, we can see some cross-section areas of the MnO_2_ nanosheets with a characteristic (001) lattice distance of 0.69 nm, which also proves that MnO_2_ crystallizes perpendicular to its (001) direction and its thickness is <10 nm. A detail lattice analysis of δ-MnO_2_, as the marked area in blue color of Figure 1E, is shown in Figure S1D. STEM-EDX mapping was used to characterize the elemental distribution of a single Co_3_O_4_@MnO_2_ nanowire, and its core@shell structure can be clearly confirmed, as shown in Figures 1F–I.

**Figure 2:**
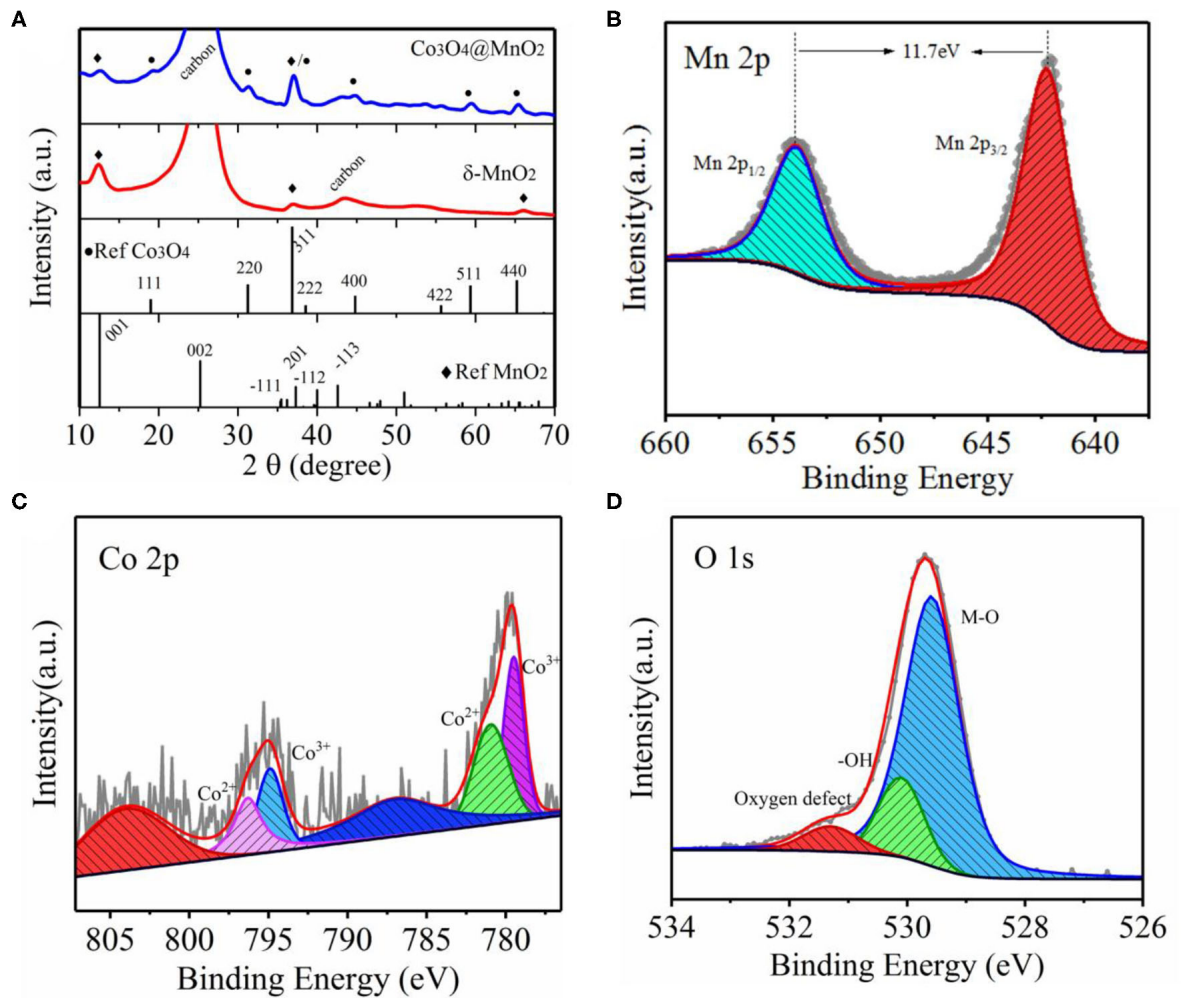
**(A)** XRD patterns of as-synthesized core-shell Co_3_O_4_@MnO_2_ and MnO_2_ on carbon cloth, respectively, and standard XRD spectra of Co_3_O_4_ and MnO_2_ as a reference. **(B–D)** High-resolution XPS spectra of Mn 2p, Co 2p, and O 1s of as-obtained Co_3_O_4_@MnO_2_, respectively.

To investigate the crystalline structure of Co_3_O_4_@MnO_2_ and its surface elemental valence states, the XRD and XPS techniques were used, as shown in Figure 2. The diffraction peaks at 19.0, 31.3, 37.0, 44.8, 59.4, and 65.4° match well with the crystal planes of (111), (220), (311), (400), (511), and (440) of Co_3_O_4_ (JCPDS 43-1003). An only rest XRD peak at 12.5° can be assigned to δ-MnO_2_ (JCPDS 80-1098), to confirm that we prepared MnO_2_ nanosheets (see SEM image of Figure S1C) on carbon cloth without pre-growing of Co_3_O_4_ nanowires. The corresponding XRD spectrum of MnO_2_ on carbon cloth is also shown in Figure 2A. The diffraction peaks at 12.5, 37.0, and 65.4° belong to (001), (−111), and (020) lattices of δ-MnO_2_ with layered structure, which is consistent with the HRTEM results. High-resolution XPS spectra of Mn 2p, Co 2p, and O 1s of as-synthesized Co_3_O_4_@MnO_2_ are shown in Figures 2B–D, respectively. The Mn 2p spectrum of Figure 2B displays two peaks at the binding energies of 642.3 and 654.0 eV, corresponding to Mn 2p 3/2 and Mn 2p 1/2 with a spin-energy separation of 11.7 eV, which indicates that Mn has 4+ valence state. The Co peaks can be assigned to Co^3+^ and Co^4+^ based on XPS analysis, which is consistent with the formation of Co_3_O_4_. The noisy XPS spectrum of Co is due to weak signal and Co is in the bulk of Co_3_O_4_@MnO_2_ but XPS is a surface technique. The O 1s spectrum in Figure 2D shows three oxygen peaks, where the Mn–O bond is at 529.8 eV, –OH is at 530.1 eV, and oxygen defect peak is at 531.3 eV. The XPS spectra of Co_3_O_4_@MnO_2_ show the characteristic photoelectron peaks of Mn 2p, Co 2p, and O 1s, and no impurities were detected.

**Figure 3:**
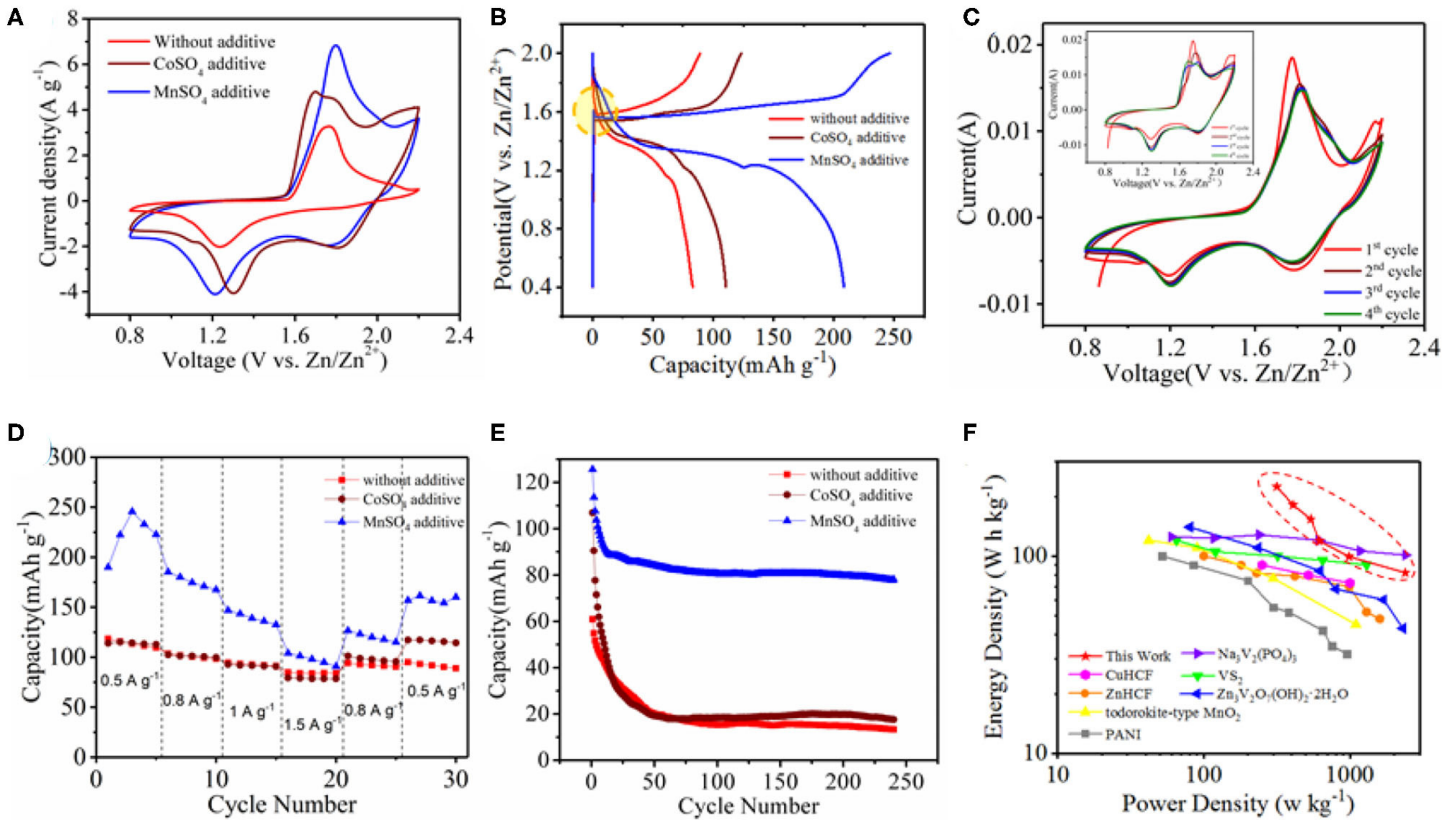
Electrochemical performance of Co_3_O_4_@MnO_2_ in 2 M ZnSO_4_ electrolyte and with CoSO_4_ or MnSO_4_ additive. **(A)** CV curves. **(B)** GCD curves. **(C)** The first four CV cycles in MnSO_4_ contained electrolyte, and within CoSO_4_ contained electrolyte (inset). **(D)** Rate performance at different current densities from 0.5 to 1.5 A g^−1^. **(E)** Cycling performance of Co_3_O_4_@MnO_2_ at 3 A g^−1^. **(F)** Ragone plots of Zn ion battery and results from literature.

We constructed a Zn ion battery device with as-synthesized Co_3_O_4_@MnO_2_ as the cathode, Zn metal as the anode, and 2.0 M ZnSO_4_ as the electrolyte, namely, Co_3_O_4_@MnO_2_//Zn. Besides, 0.2 M MnSO_4_ or CoSO_4_ is used as additive to the electrolyte to optimize the electrochemical performance of the device. Figure 3A shows CV curves of Co_3_O_4_@MnO_2_//Zn in the electrolyte with/without a cation additive. When an additive (Mn^2+^ or Co^2+^) is present, a new reduction peak appears at 1.8 V, and a new oxidation peak appears around 2.2 V, results in enlarged area of CV curves, and thus enhances energy storage capacities as compared to that without additive. We believe that it related to the electronegativity of these metal ions. As we know, Zn^2+^ will bind six water molecules in aqueous solution to form Zn[(H_2_O)_6_]^2+^ (Lee et al., 2015; Wang et al., 2018). This strong interaction disassociates H_2_O, so the pH of 2 M ZnSO_4_ solution is 4.57. When Co^2+^ or Mn^2+^ is added, disassociation of H_2_O becomes more apparent (Li G. et al., 2020). When 0.2 M CoSO_4_ or MnSO_4_ is mixed with 2 M ZnSO_4_, the solution pH value would decrease to 3.84 and 3.48, respectively. Figure 3B shows the charge and discharge curves of the Co_3_O_4_@MnO_2_ at a current density of 0.5 A g^−1^. CV curves of single pieces of MnO_2_/CC, Co_3_O_4_/CC, and Co_3_O_4_@MnO_2_/CC electrolyte were measured, and the results are shown in Figure S2. Co_3_O_4_ exhibits good electrical conductivity, indicating that it can be used as a suitable channel for electron transmission. There is a concave at around 1.6 V during charging process when the electrolyte is with Mn^2+^ additive. We experimentally found that in the Mn^2+^-containing electrolyte, the CV curve of the device became stable after the second cycle, which means Mn^2+^ can also enhance the stability of our battery device, as shown in Figure 3C. As a comparison, the CV curve was not stable after four CV cycles in the Co^2+^-containing electrolyte (Figure 3C, inset). To reveal the working mechanism of Mn^2+^ additive in our battery system, we construct a battery device of CC//Zn and performed SEM and XRD analyses of the cathode material after CV cycling in the electrolyte with and without Mn^2+^ additive, as shown in Figures S3A–C. We found that in the Mn^2+^-containing electrolyte, Mn_3_O_4_ can be detected on CC cathode by XRD analysis, as well as surface deposition happens by SEM images. Then, we can logically draw a conclusion that in the Co_3_O_4_@MnO_2_//Zn device, Mn^2+^ cations in the electrolyte can be *in situ* deposited on the cathode as Mn_3_O_4_ during electrochemical operations, which serve as electroactive cathode materials and enhance both the capacity and stability of our device. To investigate the influence of MnSO_4_ additive, electrochemical impedance spectra (EIS) measurements of Co_3_O_4_@MnO_2_ without electrolyte additives and with MnSO_4_ additive were also taken. As is shown in Nyquist plots in Figure S4, the behaviors of two EISs in the different electrolyte circumstance are quite similar. However, after examination of the EISs, we found that the charge transfer resistance of Co_3_O_4_@MnO_2_ in the Mn^2+^-doped ZnSO_4_ electrolyte (33.47 Ω) is smaller than that in the pure ZnSO_4_ electrolyte (37.32 Ω), illustrating a lower charge transfer resistance of Co_3_O_4_@MnO_2_ in the Mn^2+^-doped ZnSO_4_ electrolyte. This may account for higher ionic conductivity of the Mn^2+^-doped ZnSO_4_ electrolyte. The specific capacity and cycling stability of Co_3_O_4_@MnO_2_//Zn in three different electrolytes at different current densities from 0.5 to 1.5 A g^−1^ are shown in Figures 3D,E, respectively. We can see that the Co_3_O_4_@MnO_2_//Zn battery device has higher capacity and higher stability in the Mn^2+^ additive electrolyte. The energy density of Co_3_O_4_@MnO_2_/CC in the Mn^2+^ additive electrolyte is as high as 212.8 ± 8.2 W h kg^−1^, when the power density is 313.3 ± 1.8 W kg^−1^. The energy density is still 82.1 ± 0.3 W h kg^−1^, when the power density is 2384.7 ± 3.6 W kg^−1^. With these advantages, the energy density and power density of zinc ion batteries in this system can be much higher than that of cathode materials in reported systems. For comparison, the properties of CuHCF (Trocoli and La Mantia, 2015), ZnHCF (Zhang et al., 2015), todorokite-type MnO_2_ (Lee et al., 2013), PANI (Guerfi et al., 2014), Na_3_V_2_(PO_4_)_3_ (Hu et al., 2019), VS_2_ (He et al., 2017), and Zn_3_V_2_O_7_(OH)_2_·2H_2_O (Xia et al., 2018) are illustrated in Figure 3F. However, the Co^2+^ additive in the electrolyte does not have performance enhancement properties as compared to Mn^2+^, as shown in Figure S5.

**Figure 4:**
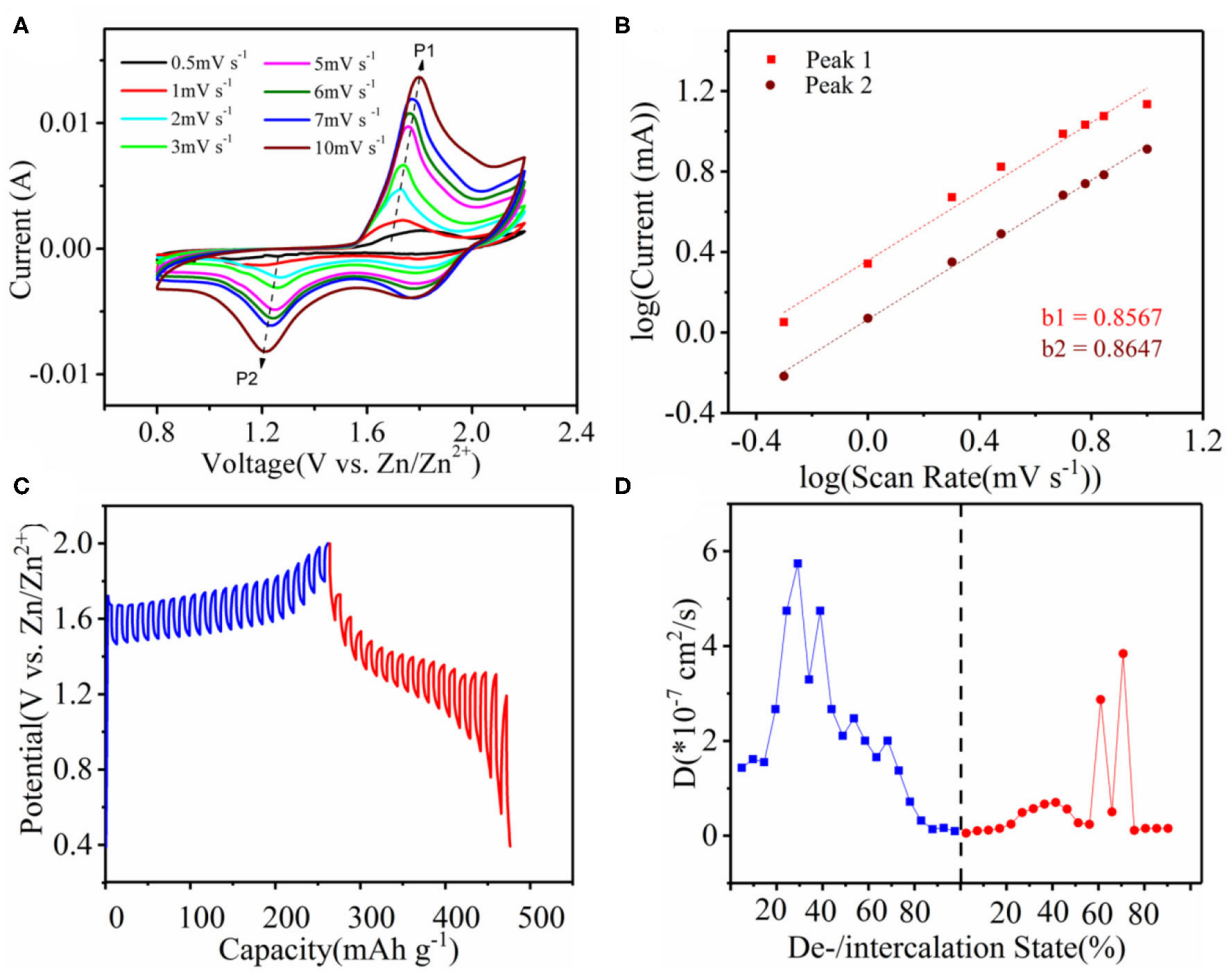
**(A)** CV behavior of Co_3_O_4_@MnO_2_ in the Mn^2+^-containing ZnSO_4_ electrolyte at different scan rates of 0.5, 1.0, 2.0, 3.0, 5.0, 6.0, 7.0, and 10.0 mV s^−1^, respectively. **(B)** Plots of log *i* vs. log *v* of the oxidation peak of P1, and reduction peak of P2, as marked in **(A)**. **(C)** GITT curves at 0.5 A g^−1^ current density. **(D)** The diffusion coefficients calculated from GITT curve.

To investigate the charge storage mechanism and dynamics of our device, we carried out electrochemical property characterization as shown in Figure 4. Figure 4A shows the CV curves of the Co_3_O_4_@MnO_2_ cathode at different scanning rates between 0.5 and 10.0 mV s^−1^. Two pairs of redox peaks in the CV curves can be clearly seen, which are consistent with the charging and discharging platforms in GCD curves. The capacitance of the battery system can be calculated by Equations (1, 2) (Tang et al., 2020):

(1) $$\begin{array}{l} I=av^{b} \end{array}$$

(2) $$\begin{array}{l} \log I=b\times \log v+\log a \end{array}$$

where *I* is the current density, *v* is the scanning rate, and *a* and *b* are adjustable parameters. When the *b*-value approaches 1.0, a capacitive response is observed. According to the graph of log *I* and log *v*, the *b*-values of peaks one and two are 0.8567 and 0.8647, respectively, indicating that the charge storage process is controlled cooperatively by the capacitance and diffusion behavior, which leads to fast Zn^2+^ diffusion dynamics (Figure 4B). The contribution of the capacitor can be separated by quantifying the current (*I*) at a fixed potential (*V*), the capacitance effect (*k*_1_*v*), and the diffusion-controlled insertion (*k*_2_*v*^1/2^), according to the following Equation (3)

(3) $$\begin{array}{l} I=k_{1}v+k_{2}v^{1/2} \end{array}$$

Figure S6A depicts a typical 10 mV s^−1^ capacitive current (blue area) compared to the total current. About 95.4% of the total charge comes from the capacitive contribution, which is closely related to the battery's rate performance. We further quantified the non-diffusion control capability of the Co_3_O_4_@MnO_2_ cathode, as shown in Figure S6B. When the scanning rate was 0.5, 1, 2, 3, 5, 6, 7, and 10 mV s^−1^, the contribution rate of the capacitor control is 39.4, 53.6, 63.0, 70.3, 77.0, 80.7, 82.9, and 95.4%, respectively. This is why the Co_3_O_4_@MnO_2_/CC punch type cell has such a good rate performance and a high power density along with a high energy density. According to the result of galvanostatic intermittent titration technique (GITT) in Figure 4C, the diffusion coefficient (*D*) is obtained from the following Equation (4) (Yang and Rogach, 2019):

(4) $$\begin{array}{l} D=\frac{4}{\pi \tau }{\left(\frac{m_{B}V_{M}}{M_{B}S}\right)}^{2}{\left(\frac{△E_{S}}{△E_{\tau }}\right)}^{2} \end{array}$$

where τ is a constant current pulse time; *m*_*B*_, *V*_*M*_, *S*, and *M*_*B*_ are the mass loading, the molar volume, the electrochemical active area, and the molar weight, respectively; and *E*_*S*_ and *E*_τ_ are the voltage changes in the rest and dis-/charge steps. On the basis of GITT results, we calculated the diffusion coefficients at each point as is shown in Figure 4D. The diffusion coefficient values are all <6 × 10^−7^ cm^2^ s^−1^.

**Figure 5:**
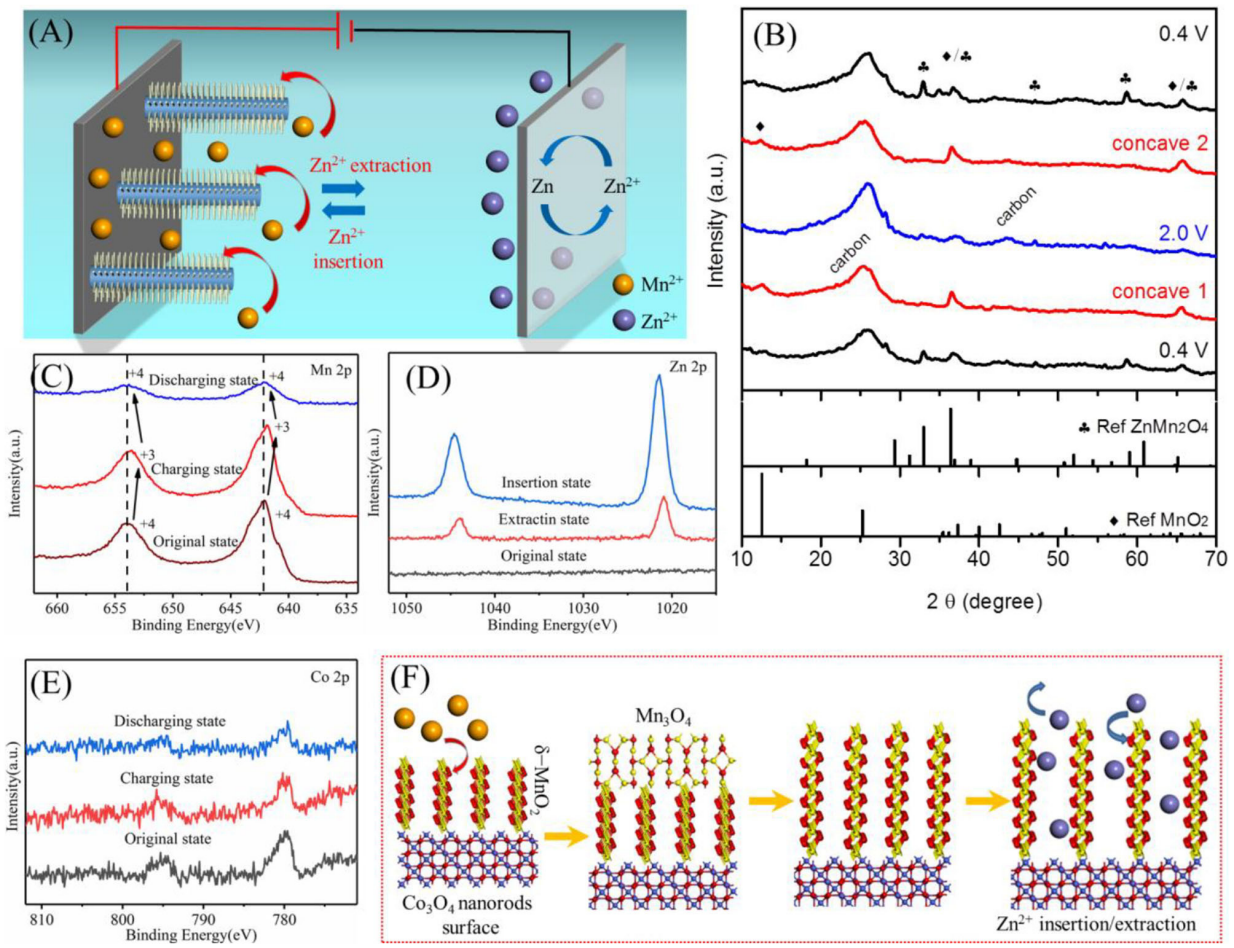
**(A)** Illustration of the working mechanism of the Zn ion battery device. **(B)** XRD curves of MnO_2_/CC at different charge/discharge states. **(C–E)** XPS spectra of Mn 2p, Zn 2p, and Co 2p at different charge/discharge states. **(F)** Summary of the electrolyte contribution to an as-synthesized Co_3_O_4_@MnO_2_ cathode in our battery system and the electrochemical energy storage mechanism of the cathode.

The typical chemistry of zinc ion batteries is Zn^2+^ insertion/extraction into/out of the cathode materials, accompanied by Zn^2+^ ↔ Zn Faradic reaction at the anode. Among which, the cathode part is critical but rarely studied, meanwhile, cation additives of electrolytes could enhance system complexity. We proposed a working mechanism of our zinc ion battery with the Co_3_O_4_@MnO_2_/CC cathode and the Mn^2+^-containing electrolyte, as shown in Figure 5A. To clarify that, we use XRD and XPS to analyze MnO_2_/CC cathode at different charge/discharge states, due to MnO_2_ serving as electroactive materials in our system and its noisy or confusing XRD spectrum when Co_3_O_4_ is present (see Figure 2A). Figure 5B shows that the XRD spectra of MnO_2_/CC started at 0.4 V (bottom curve) and then charged into concave one (~1.6 V) and 2.0 V, followed by a discharge process to concave two (~1.6 V) and 0.4 V. The XRD peaks are mainly assigned to ZnMn_2_O_4_ and MnO_2_. During a charging process from bottom 0.4 V to 2.0 V, Zn^2+^ cations were gradually extracted out of ZnMn_2_O_4_, resulting in a decay of ZnMn_2_O_4_ peaks and an increase of MnO_2_ peaks, as shown in Equation (5).

(5) $$\begin{array}{l} \text{Z}{\text{n}}_{\text{x}}\text{Mn}{\text{O}}_{2}\to \text{Mn}{\text{O}}_{2}+\text{xZ}{\text{n}}^{2+}+2\text{x}{\text{e}}^{-} \end{array}$$

The XRD peaks for ZnMn_2_O_4_ is thus the weakest at 2.0 V of the blue XRD spectrum. Following a discharge process, Zn^2+^ cations were inserted into the cathode material, forming the Zn_*x*_MnO_2_ phase, as shown in Equation (6).

(6) $$\begin{array}{l} \text{Mn}{\text{O}}_{2}+\text{xZ}{\text{n}}^{2+}+2\text{x}{\text{e}}^{-}\to \text{Z}{\text{n}}_{\text{x}}\text{Mn}{\text{O}}_{2} \end{array}$$

The XRD peaks for ZnMn_2_O_4_ is then recovered when discharged to 0.4 V again, as compared with black XRD spectra, which demonstrates high reversibility of Zn^2+^ extraction/insertion of the cathode materials during charging and discharging. Figure 5C shows high-resolution Mn XPS spectra of MnO_2_/CC at different charge/discharge states, revealing the change of Mn valence from mainly 4+ to mainly 3+ and then back to mainly 4+. During charging, Mn_3_O_4_ was deposited on the electrode surface following Equation (7), resulting in 3+ dominated XPS peak (Hao et al., 2018).

(7) $$\begin{array}{l} 3\text{M}{\text{n}}^{2+}+4{\text{H}}_{2}\text{O}\to \text{M}{\text{n}}_{3}{\text{O}}_{4}+8{\text{H}}^{+}+2{\text{e}}^{-} \end{array}$$

After discharging, Mn_3_O_4_ is oxidated into *MnO*_2_ according to Equation.

(8) $$\begin{array}{l} \text{M}{\text{n}}_{3}{\text{O}}_{4}+\text{x}{\text{H}}_{2}\text{O}\to 2\text{Mn}{\text{O}}_{2}\cdot \text{x}{\text{H}}_{2}\text{O}+\text{M}{\text{n}}^{2+}+2{\text{e}}^{-} \end{array}$$

Figures S7A,B show SEM-EDS results of single Co_3_O_4_@MnO_2_ nanorods after firstly being discharged to 0.4 V and then followed by being charged to 2.0 V, respectively. The change of the Zn content in Figures S7A,B and the change of XPS peak intensity of Zn in Figure 5D prove the inserting/extracting Zn^2+^. XPS results of Figure 5E show that the binding energy of Co does not change much, which indicates that Co_3_O_4_ does not take part in electrochemical reactions. Based on the above analysis, the working mechanism of our cathode can be summarized in Figure 5F (Cheng et al., 2016; Shi et al., 2020). Many studies have also found that Mn_3_O_4_ has a tendency of phase transition to a layered MnO_2_ in the cycling process (Jabeen et al., 2017; Hao et al., 2018). In this paper, after discharge, the surface of Mn_3_O_4_ will undergo a phase transition. Mn^2+^ will continue to dissolve into the electrolyte, and Mn^3+^ oxidizes to Mn^4+^, becoming layered MnO_2_. The resulting layered manganese dioxide and the previously coated manganese dioxide jointly participate in the insertion and extraction of zinc ions, and the 0.69-nm layer spacing enables a good insertion process of Zn ions.

## Conclusions

In conclusion, a core@shell Co_3_O_4_@MnO_2_ nanowire has been synthesized on carbon cloth by a two-step hydrothermal method. The as-synthesized MnO_2_ is a layered δ-MnO_2_ phase and consists of nanosheets. When used as a cathode of an aqueous Zn ion battery with Mn^2+^ additive in a ZnSO_4_ electrolyte, the core@shell Co_3_O_4_@MnO_2_ battery presents a high energy density of 212.8 ± 8.2 Wh kg^−1^ at a power density of 313.3 ± 1.8 W kg^−1^ and maintains 82.1 ± 0.3 Wh kg^−1^ at a power density of 2384.7 ± 3.6 W kg^−1^, which is much higher than that for a pristine ZnSO_4_ electrolyte or for the electrolyte with a Co^2+^ additive. In addition, a ZIB with a high output voltage of 2.0 V is presented. The performance enhancement mechanism of the Mn^2+^ additive in the electrolyte is that Mn^2+^ can be electrochemically oxidized to Mn_3_O_4_ on the cathode and further converted to electroactive MnO_2_. The electrochemistry of our Zn ion battery reveals reversible Zn cation and proton insertion/extraction into/out of layered δ-MnO_2_. This work demonstrates a promising cathode for Zn ion battery and other electrochemical energy storage devices.

## Data Availability Statement

All datasets generated for this study are included in the article/Supplementary Material.

## Author Contributions

NW and GY carried out the experimental parts of materials synthesis and characterizations. NW wrote the manuscript. GY, CW, QT, JJ, and XZ assisted the experiments and took part in data analysis and discussions on the results. XC, HaoW, JZ, XP, and Hwan were involved in designing and supervising this project and they have made a great contribution to the discussions. XC, YW, and PA carried out TEM characterization, analyzed TEM data, and played an important role in interpreting the results. HanW supervised the project and contributed to writing the paper, conceived and designed the battery device, and analyzed all the experiment data. All co-authors contributed to commenting on, editing the manuscript and SI. All authors contributed to the article, and approved the submitted version.

## Conflict of Interest

The authors declare that the research was conducted in the absence of any commercial or financial relationships that could be construed as a potential conflict of interest.
