# Supplementary material for: Contribution of Cation Addition to MnO2 Nanosheets on Stable Co3O4 Nanowires for Aqueous Zinc-Ion Battery
Source: Front Chem. 2020 Sep 23;8:793. doi: 10.3389/fchem.2020.00793 (PMC7539680; doi:10.3389/fchem.2020.00793)
Supplement: Supplementary file 1 [file Table_1.docx]

**Supporting Information**

**Contribution of cation addition to MnO_2_ nanosheets on stable Co_3_O_4_ nanowires for aqueous zinc-ion battery**

Nengze Wang,^a,1^ Gaochen Yang,^a,1^ Yi Gan,^a^ Houzhao Wan,^a,^* Xu Chen,^b,^* Cong Wang,^a^ Qiuyang Tan,^a^ Jie Ji,^a^ Xiaojuan Zhao,^a^ Pengcheng Liu,^a^ Jun Zhang,^a^ Xiaoniu Peng,^a^ Hanbin Wang,^a,*^ Yi Wang,^b^ Guokun Ma,^a^ and Peter A. van Aken,^b^ and Hao Wang ^a^

^a^ Hubei Key Laboratory of Ferro & Piezoelectric Materials and Devices, Faculty of Physics and Electronic Science, Hubei University, Wuhan 430062, China.

^b^ Max Planck Institute for Solid State Research, Heisenbergstr. 1, D-70569 Stuttgart, Germany.

^1^ These author contributed equally to this work.

***** **Corresponding authors**:

Dr. Houzhao Wan, E-mail: [houzhaow@hubu.edu.cn](mailto:houzhaow@hubu.edu.cn)

Dr. Xu Chen, E-mail: [X.Chen@fkf.mpg.de](mailto:xu.chen@fkf.mpg.de)

Prof. Hanbin Wang, E-mail: [123272314@qq.com](mailto:123272314@qq.com)


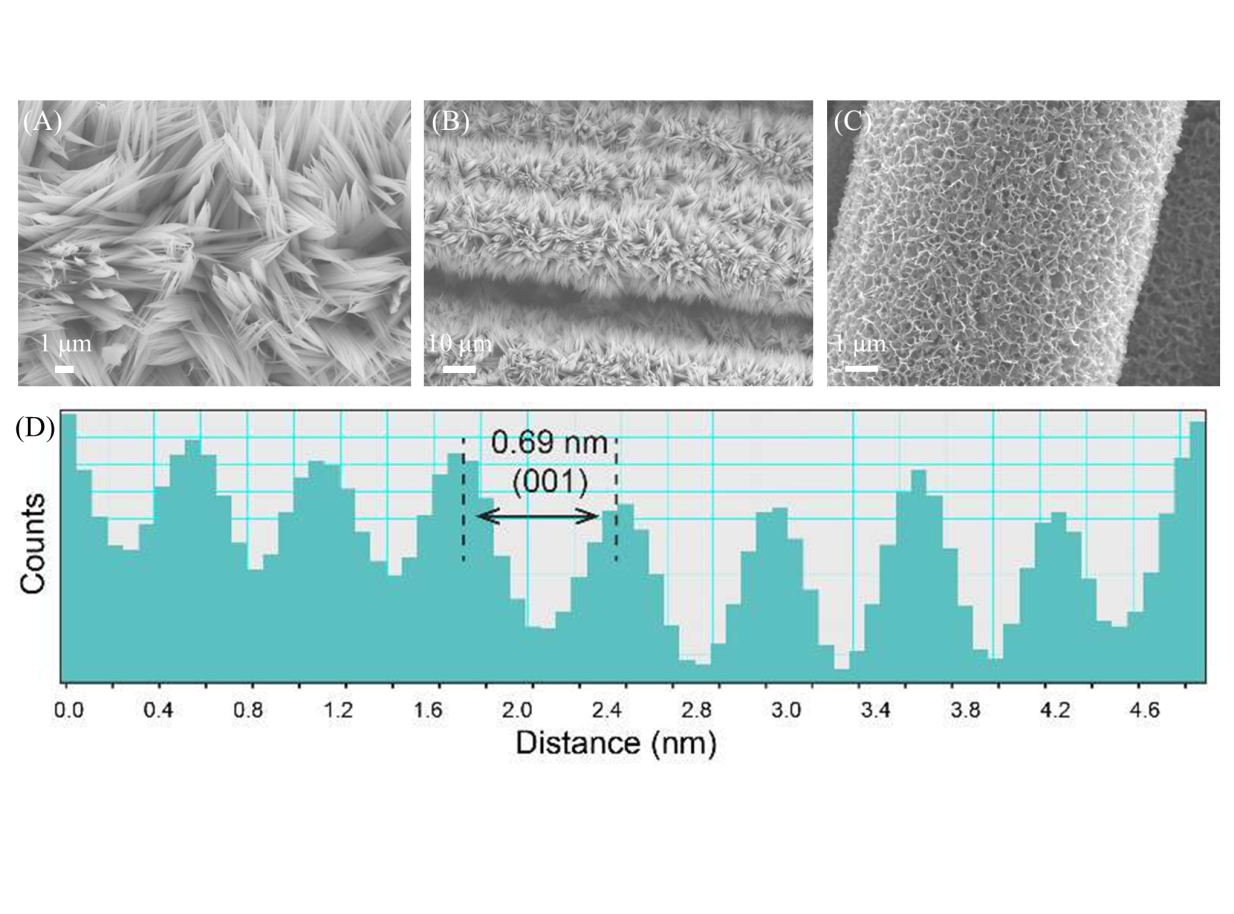


**Figure S**1 **|** **(A,B)** SEM images of Co_3_O_4_/CC. **(C)** SEM image of MnO_2_/CC. (**D**) HRTEM intensity profile of Fig. 1E.





**Figure S**2 **|** CV curves of a single piece of MnO_2_/CC, Co_3_O_4_/CC and Co_3_O_4_@MnO_2_/CC (clamped with a gasket test).


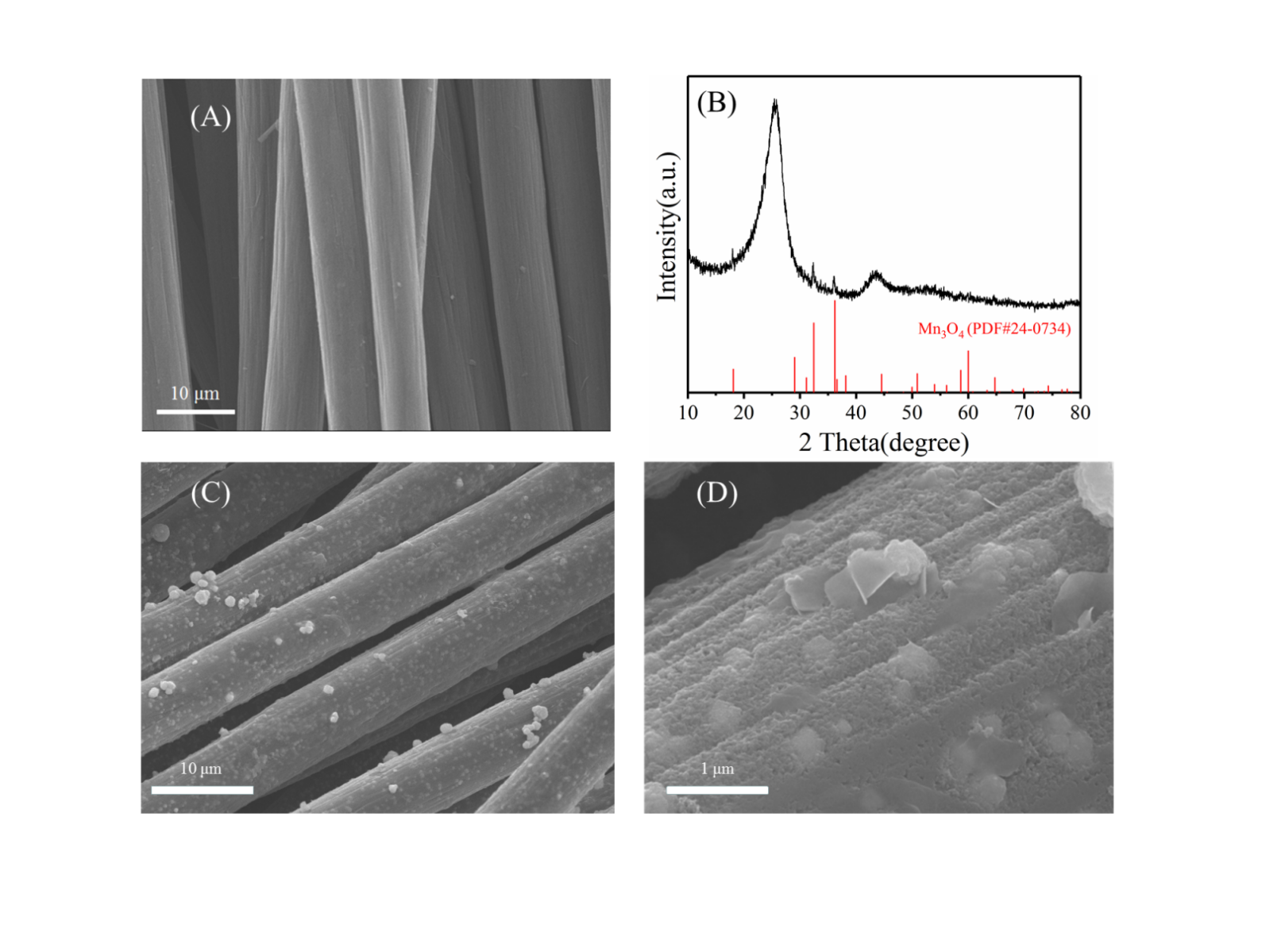


**Figure S**3 **|** **(A)** SEM image of CC charged in 2 M ZnSO_4_. **(B)** XRD profile of CC charged in 2 M ZnSO_4_ with 0.2 M MnSO_4_. **(C,D)** SEM images of CC charged in 2 M ZnSO_4_ with 0.2 M MnSO_4_.


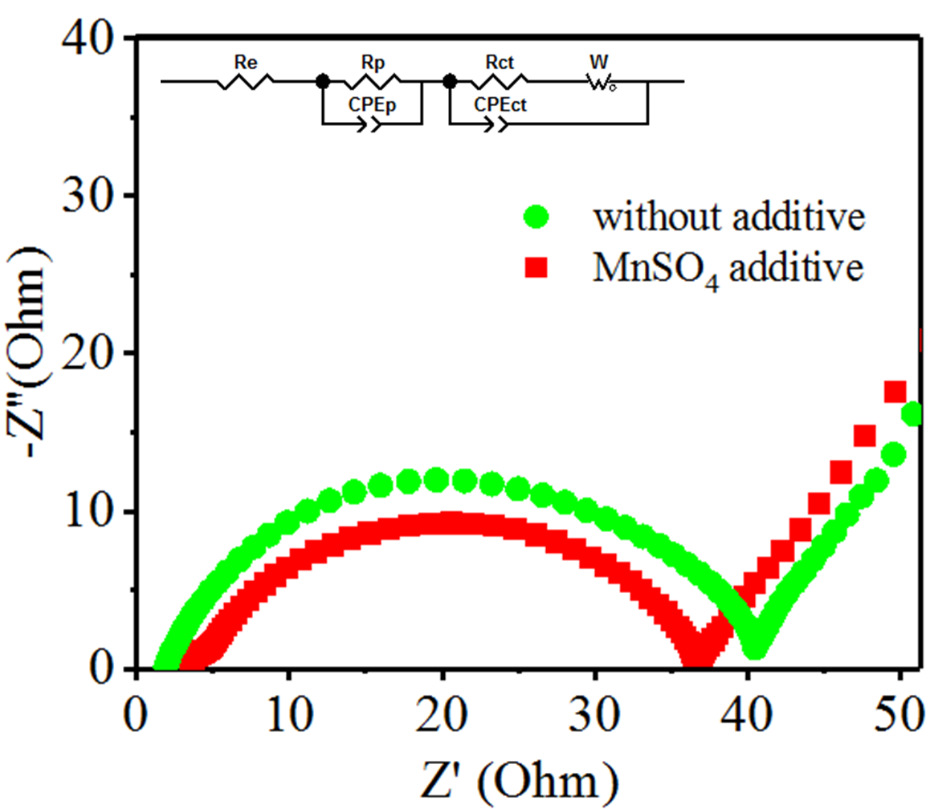


**Figure S**4 **|** Nyquist plots of our Zn//Co_3_O_4_@MnO_2_ battery in ZnSO_4_ electrolyte (green color), and in ZnSO_4_ electrolyte with Mn^2+^ additive (red color).


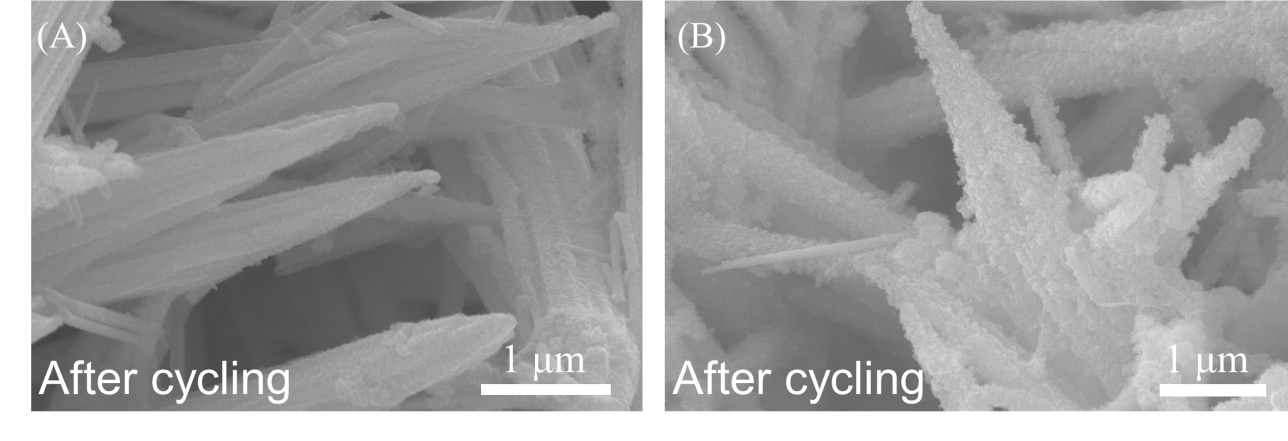


**Figure S**5 **|** **(A)** SEM image of Co_3_O_4_/CC cycled 1000 times in 2 M ZnSO_4_ with 0.2 M CoSO_4_ at 0.5 A g^−1^_._ **(B)** SEM image of Co_3_O_4_/CC cycled 1000 times in 2 M ZnSO_4_ with 0.2 M MnSO_4_ at 0.5 A g^−1^_._


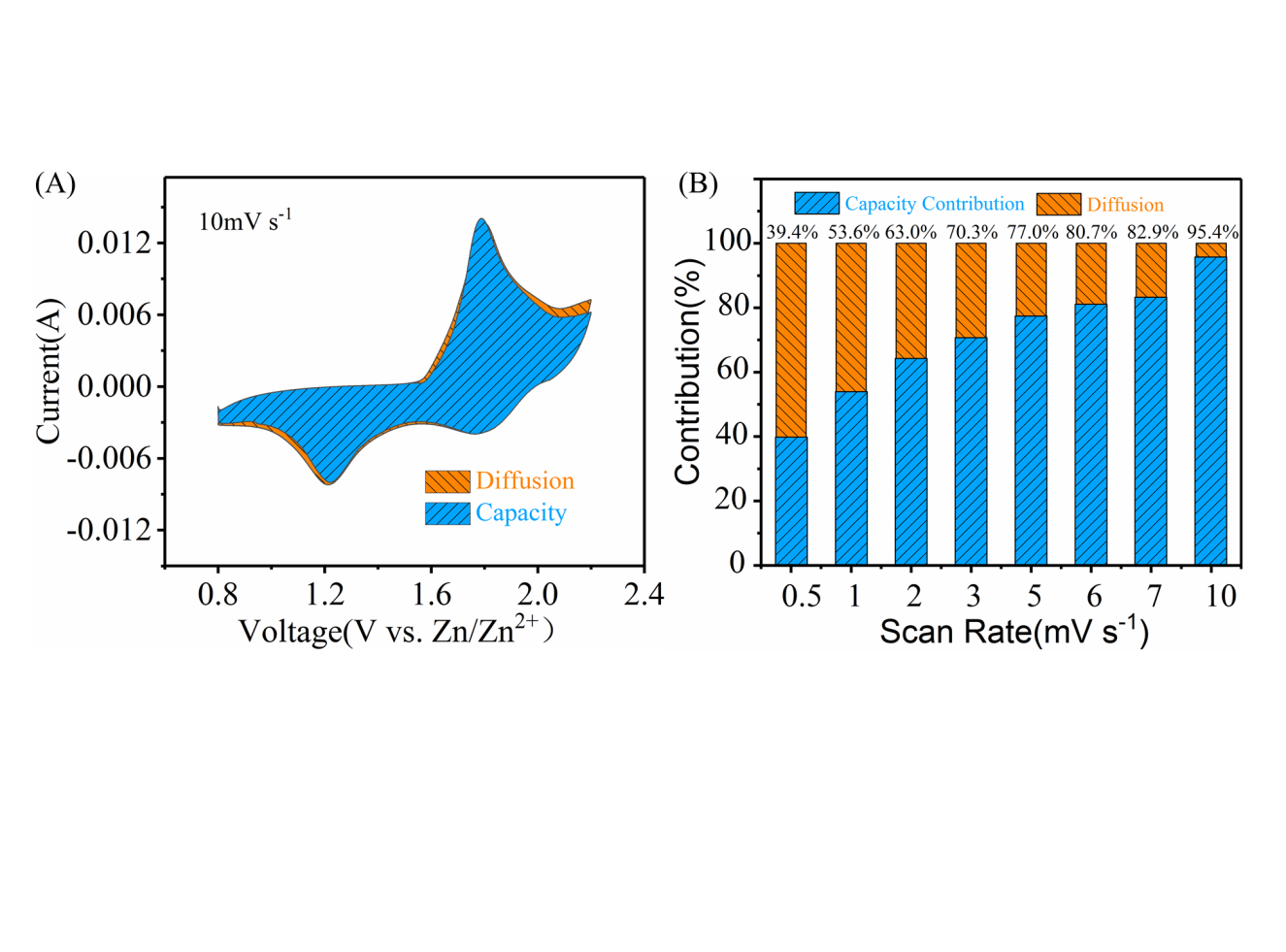


**FIGURE S**6 **|** **(A)** Capacitance contribution ratio at 10.0 mV s^−1^. **(B)** Columnar table of capacitive contribution ratios.


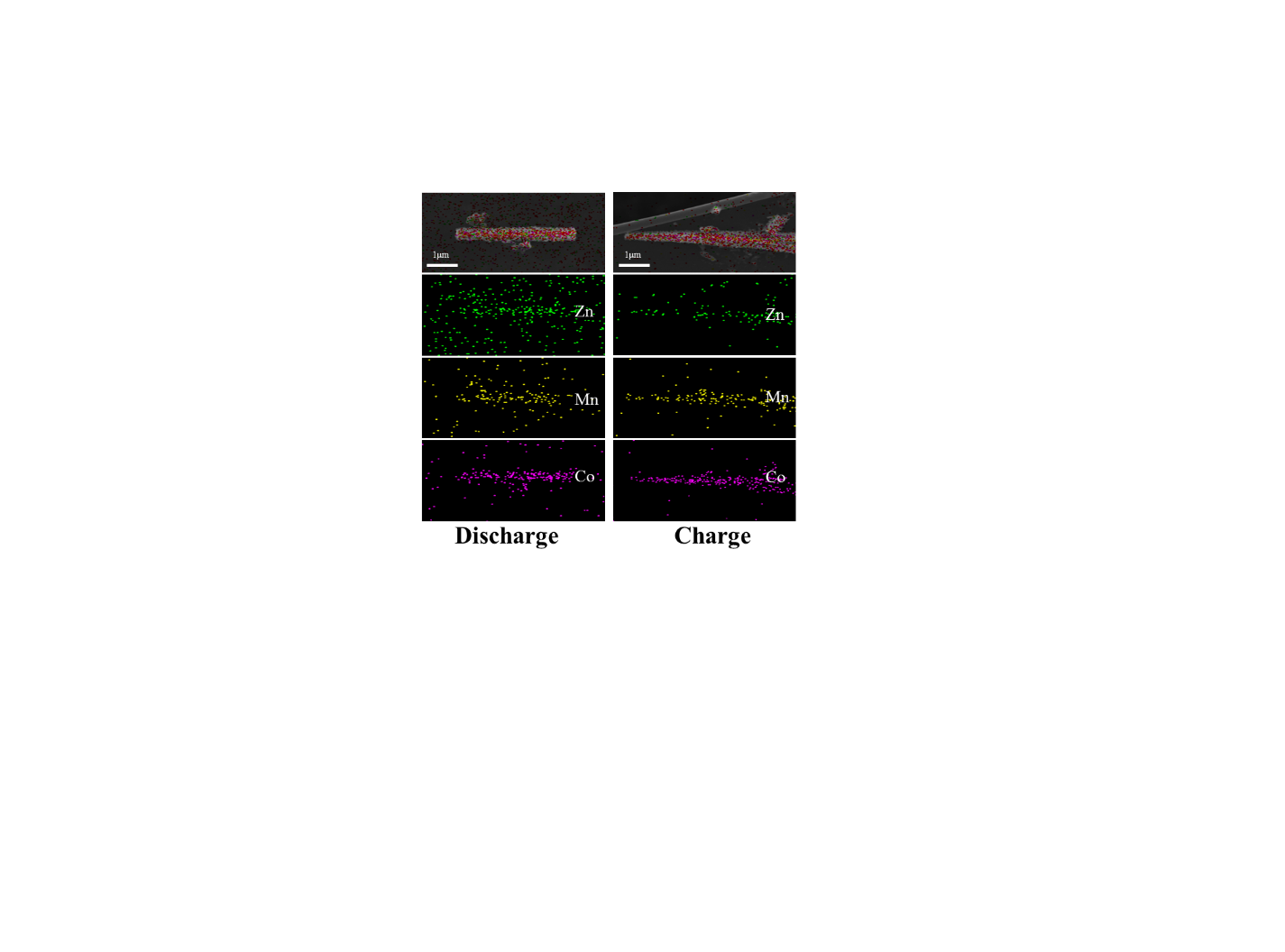


**Discharge**

**Charge**

**Figure S**7 **|** **(A,B)** SEM-EDS mapping images of the Co_3_O_4_@MnO_2_ when it was discharged to 0.4 V and then charged to 2.0 V, respectively.
